# FUT4siRNA augments the chemosensitivity of non-small cell lung cancer to cisplatin through activation of FOXO1-induced apoptosis

**DOI:** 10.1186/s12885-020-07324-z

**Published:** 2020-09-18

**Authors:** Wei Gao, Jinxiao Liang, Yiru Ye, Jinlan Lu, Tongtong Lin, Na Wang, Jingyin Dong, Jianping Pan

**Affiliations:** 1 Department of Clinical Medicine, Zhejiang University City College School of Medicine, 50 Huzhou Road, Hangzhou, 310015 P.R. China; 2 Department of Toracic Surgery, Zhejiang Cancer Hospital, Hangzhou, 310000 China

**Keywords:** FUT4, Chemosensitivity, Cisplatin, NSCLC, FOXO1

## Abstract

**Background:**

Increased fucosylation is associated with the chemoresistance phenotype. Meanwhile, fucosyltransferase IV (FUT4) amounts are frequently elevated in lung cancer and may be related to increased chemoresistance.

**Methods:**

In the present work, FUT4’s role in cisplatin-induced apoptosis was assessed in A549 and H1975 cells, respectively. To clarify whether the FUT4 gene attenuates chemosensitivity in tumor cells, we constructed FUT4siRNA and evaluated its effects on cisplatin-induced apoptosis and cell growth inhibition. Cell viability, apoptosis, migration and invasion assay were conducted to investigate cisplatin sensitivity. The activation of EGFR/AKT/FOXO1 signaling were measured by western blot. The translocation of FOXO1 was assessed by IFC using Laser Scanning Confocal Microscope.

**Results:**

We found that FUT4 knockdown dose-dependently increased cisplatin-associated cytotoxicity. Furthermore, FUT4 silencing induced apoptosis and inhibited proliferation in A549 and H1975 cells by suppressing Akt and FOXO1 phosphorylation induced by cisplatin administration, which resulted in nuclear translocation of FOXO1.

**Conclusion:**

These results suggested FUT4 might control chemoresistance to cisplatin in lung cancer by suppressing FOXO1-induced apoptosis.

## Background

As one of the main causes of mortality around the world [1], non-small cell lung cancer (NSCLC) contributes to approximately 85% of cancer-related deaths [2] and has limited treatment options [3]. Chemotherapeutics are commonly employed to treat advanced NSCLC. Cisplatin, a platinum drug, kills malignant cells and is widely applied in NSCLC [4]. However, drug resistance constitutes an important clinical challenge, hampering the application in platinum-based chemotherapy of NSCLC [5]. Therefore, deciphering the molecular mechanisms related to chemoresistance would help develop novel strategies for identifying new drug sensitizers that increase the potency of platinum drugs.

Cell-surface glycans play important roles in many physiological and pathological processes [6]. Fucose contributes to the structural composition of various carbohydrate chains. The binding of a fucosyl residue to the terminus of glycosidic chains serves as an important component of the carbohydrate structure of some essential cell surface proteins which are closely associated with carcinogenesis [7]. Fucosyltransferases (FUTs) serve as critical enzymes catalyzing the biosynthesis of fucosylated polysaccharides. FUTs catalyze L-fucose transfer from GDP-fucose to acceptor molecules; 4 types of linkage have been reported, including 1, 2-, 1, 3/4-, and 1, 6-linkages. α1,3-fucosylation of LeY is catalyzed by FUT4 [8]. In A431 cells, overexpression of FUT4 activates the PI3K/Akt signaling pathway via phosphorylation of EGFR [9]. Recent reports have shown that FUT4/LeY is closely related to multidrug resistance, and high expression of FUT4 is involved in human HCC cell drug resistance through induction of PI3K/AKT signaling [10]. In agreement, breast cancer T47D/ADR cells displaying drug resistance overexpress FUT4 [11]. Lewis Y antigen whose formation is catalyzed by FUT4 is closely associated with the regulation of many drug resistance-related proteins in the human ovarian carcinoma RMG-I-H cell line [12]. Elevated expression levels of Lewis Y and FUT4 are observed in lung cancer [13]; however, whether FUT4 affects the chemosensitivity of NSCLC remains unclear.

O-class forkhead factors (FOX) family comprises FOXO1, FOXO2, FOXO3 and FOXO4 [14]. FOXO1 plays as a key transcription factor as a member of FOXO family [15]. FOXO1 was found to be one of the most important substrate of AKT. It has been reported that the activated AKT induces phosphorylation of FOXO1 at 3 different serine/threonine residues and thus decrease the nuclear translocation of FOXO1 [16]. AKT/FOXO1 signaling pathway regulates many biological processs by modulating numerous target genes which are involved in apoptosis, autophagy and cell cycle arrest [17, 18].

Cisplatin represents one of the most potent wide-spectrum anticancer chemotherapeutics, and is frequently applied in NSCLC. However, almost all cancer types acquire resistance to cisplatin, which reduces its efficacy [19]. The present study aimed to provide evidence of the efficacy of targeting genes during cisplatin treatment in the future.

## Methods

### Cell culture

A549 and H1975 cells were provided by American Type Culture Collection (USA). DMEM/F12 (1:1), RPMI 1640, fetal bovine serum (FBS), and Lipofectamine™ were manufactured by Life technologies (USA). Cisplatin (DDP) was purchased from Zhejiang Cancer Hospital. LY294002 was provided by Selleck Chemicals (USA). Anti-FOXO1, anti-Akt, anti-PARP-1 and anti-EGFR primary antibodies were purchased from Proteintech (USA). Primary antibodies against p-Akt, p-FOXO1, p-PARP-1 and p-EGFR were from Cell Signaling Technology (USA). FUT4 small interfering RNAs (siRNAs) were constructed by GenePharma. The siRNA sequences of FUT4 were:

5′-GATCCGCCTGGCAAGTAACCTCTTCTCAAGAGAAAGAGGTTACTTGCCAGGCTTA-3′ and 5′-AGCTTAAAGCCTGGCAAGTAACCTCTTTCTCTTGAGAAGAGGTTACTTGCCAGGCG-3′.

### Cell culture

A549 and H1975 cells were cultured in DMEM/F12 (1:1) containing 10% heat-treated FBS, 100 U/ml penicillin and 50 mg/ml streptomycin, at 37 °C in a humid environment with 5% CO2. The cells were free from mycoplasma contamination.

### Small interfering RNA treatment

A549 and H1975 cells were submitted to reverse-transfection with siRNAs (at 10 nM) employing Lipofectamine 2000 (Invitrogen) as instructed by the manufacturer. Upon incubating of cells with medium containing siRNAs for 48 h, the transfected cells were collected and used in subsequent analyses.

### Flow cytometry

Cell apoptosis was assessed with an Annexin V-FITC/propidium iodide (PI) double staining kit as directed by the manufacturer. Briefly, A549 and H1975 cells seeded in 6-well plates, respectively, were incubated overnight for attachment and transfected with FUT4siRNA. Then, cisplatin (3 μg/ml) in medium was supplemented for 48-h incubation. Harvested cells were submitted to two washing steps with chilled PBS, resuspended in 250 μl of binding buffer and stained with Annexin V/FITC-PI for half an hour away from light. Finally, cells were assessed on a FACS Calibur flow cytometer (BD Biosciences, USA).

### Western blot

Cells were lysed with the radio-immunoprecipitation assay (RIPA) buffer containing protease inhibitors (Selleck, USA). The lysates were centrifuged for 10 min at 4 °C, and the resulting supernatants were assessed for total protein amounts with Enhanced BCA Protein Assay Kit (Beyotime, China). Equal amounts of total protein (50 μg) were resolved by SDS polyacrylamide gel electrophoresis (SDS-PAGE) and electro-transferred onto nitrocellulose membranes. After blocking (5% nonfat milk in TBS/Tween 20; 0.05%, v/v) for 1 h at RT, the samples were incubated with primary antibodies (1:200–1:2000) targeting FUT4, LeY, Akt, pAkt, FOXO1, EGFR, pEGFR, and GAPDH, respectively. This was followed by incubation with HRP-linked secondary antibodies for detection.

### Immunofluorescence

A549 and H1975 cells cultured on glass coverslips were submitted to fixation with 4% paraformaldehyde (30 min) and Triton X-100 treatment (0.1%; 10 min, 4 °C). Goat serum was used for blocking (1 h, 37 °C), and the cells were incubated with rabbit anti-FOXO1 (1:100) at 4 °C overnight. The cells were then washed with cold PBS 3 times and incubated with FITC-linked goat anti-rabbit secondary antibodies (1 h, RT). After counterstaining with DAPI (1 μg/ml) for 10 min, images were captured on an Olympus confocal microscope at 200 × (CLSM, FV1000, Olympus, Tokyo, Japan), equipped with a band-path filter set (Olympus). The emission signal was recorded with a CCD camera. The fluorescent signals were recorded and analyzed using Olympus-analyzer software (FV10-ASW3.0 Viewer).

### Colony formation assay

Treated cells in 6-well plates (200 cells/well at seeding) were incubated for 2 weeks, and colonies were submitted to staining with 0.05% crystal violet upon methanol fixation.

### Invasion assay

Cells (1 × 10^5^/well) in 200 μL of serum-free DMEM/F12 medium were seeded in the upper chambers containing Matrigel-coated membranes. In the lower chambers, 800 μL of DMEM/F12 medium containing 10% FBS was added. The cells were incubated for 24 h at 37 °C in a humid environment containing 5% CO2. Upon fixation with 100% methanol (20 min) and staining with 0.1% crystal violet (15 min), image acquisition was performed on an Olympus BX83 fluorescence microscope (Olympus).

### Wound healing assay

A549 and H1975 cells were transfected with fut4siRNA and/or cisplatin (3 g/ml) treatment. Then the cells were seeded in 6-well plate for 24 h. The monolayers were scratched with a 200 μl pipette tip, followed by washing with serum free DMEM/F12 medium to remove the detached cells. The wounded areas were observed and imaged under microscope after 24 h.

### CCK-8 assay

Viable cells were assessed by CCK-8 (Dojindo Laboratories, Japan) as directed by the manufacturer. A549 and H1975 cells (6 × 10^3^/90 μL/well) were seeded in 96-well plates, incubated for 24 h and transfected with FUT4siRNA. This was followed by administration of cisplatin (0, 0.6, 1.2, 2.5, 5, 10, 20 and 40 μg/ml, respectively) for 24-h incubation. Optical density was obtained at 450 nm on a microplate reader (Thermo Fisher, USA).

### Statistical analysis

Values are mean ± SD from triplicate experiments carried out three times or more. Groups were compared by Student’s t-test or one way analysis of variance (ANOVA) and Tukey’s post hoc analysis. and *p* < 0.05 was deemed statistically significant.

## Results

### Downregulation of FUT4 inhibits the expression of FUT4 as well as Lewis Y (LeY)

**Fig. 1:**
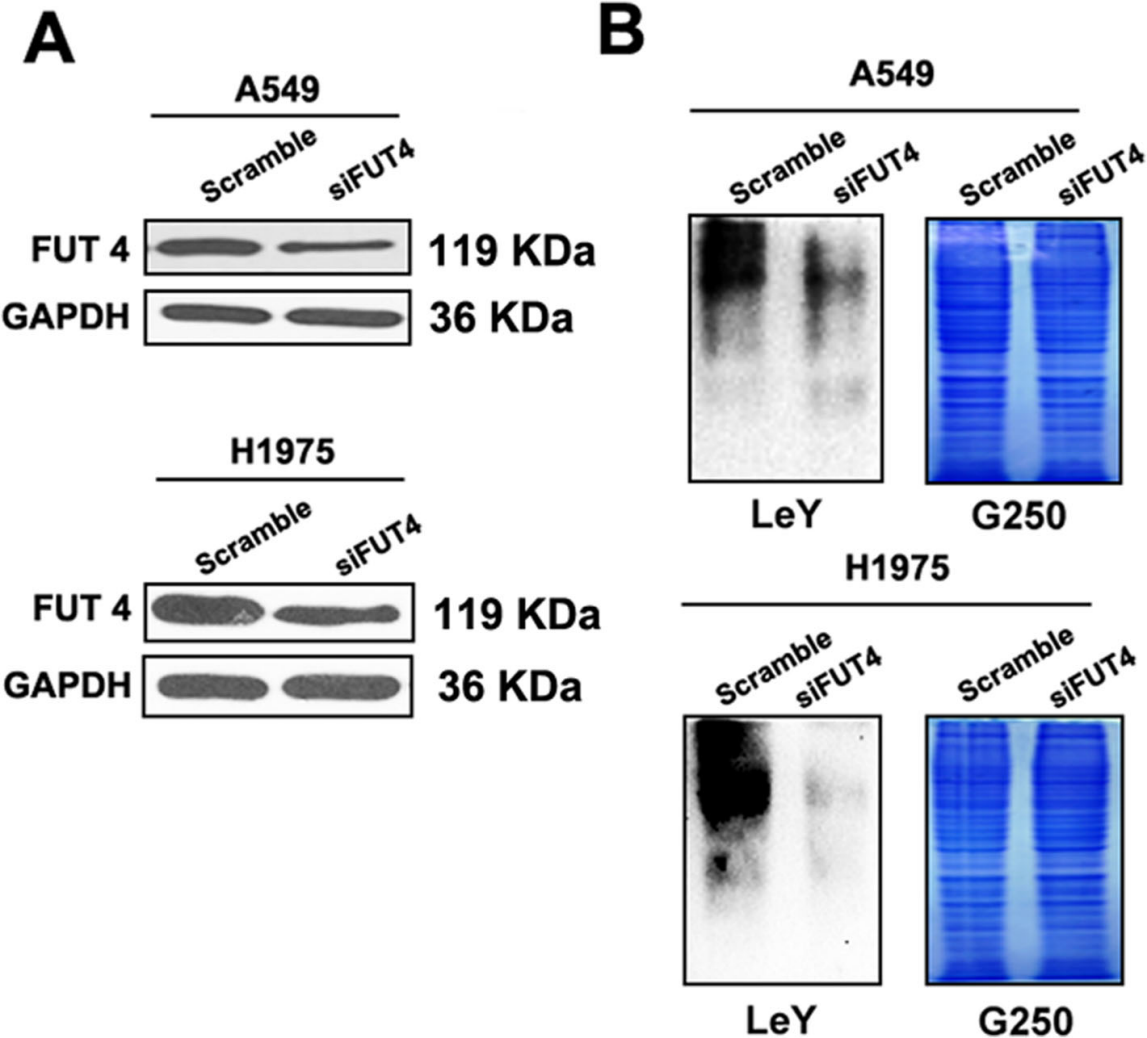
FUT4siRNA transfection downregulates FUT4 and Lewis Y. A549 and H1975 cells were transfected with FUT4siRNA. **a** Western blot was employed to examine FUT4 protein expression. **b** Western blot was employed to assess Lewis Y expression. Coomassie brilliant blue (G250) staining of gels shows comparable amounts of protein in each lane. The results are representative of three separate triplicate experiments

Western blot was performed to examine whether FUT4siRNA regulates the expression of Lewis Y antigen (Fig. 1). It was demonstrated that FUT4siRNA significantly reduced FUT4 and Lewis Y levels in both A549 and H1975 cells.

### FUT4 silencing sensitizes A549 and H1975 cells to cisplatin

**Fig. 2:**
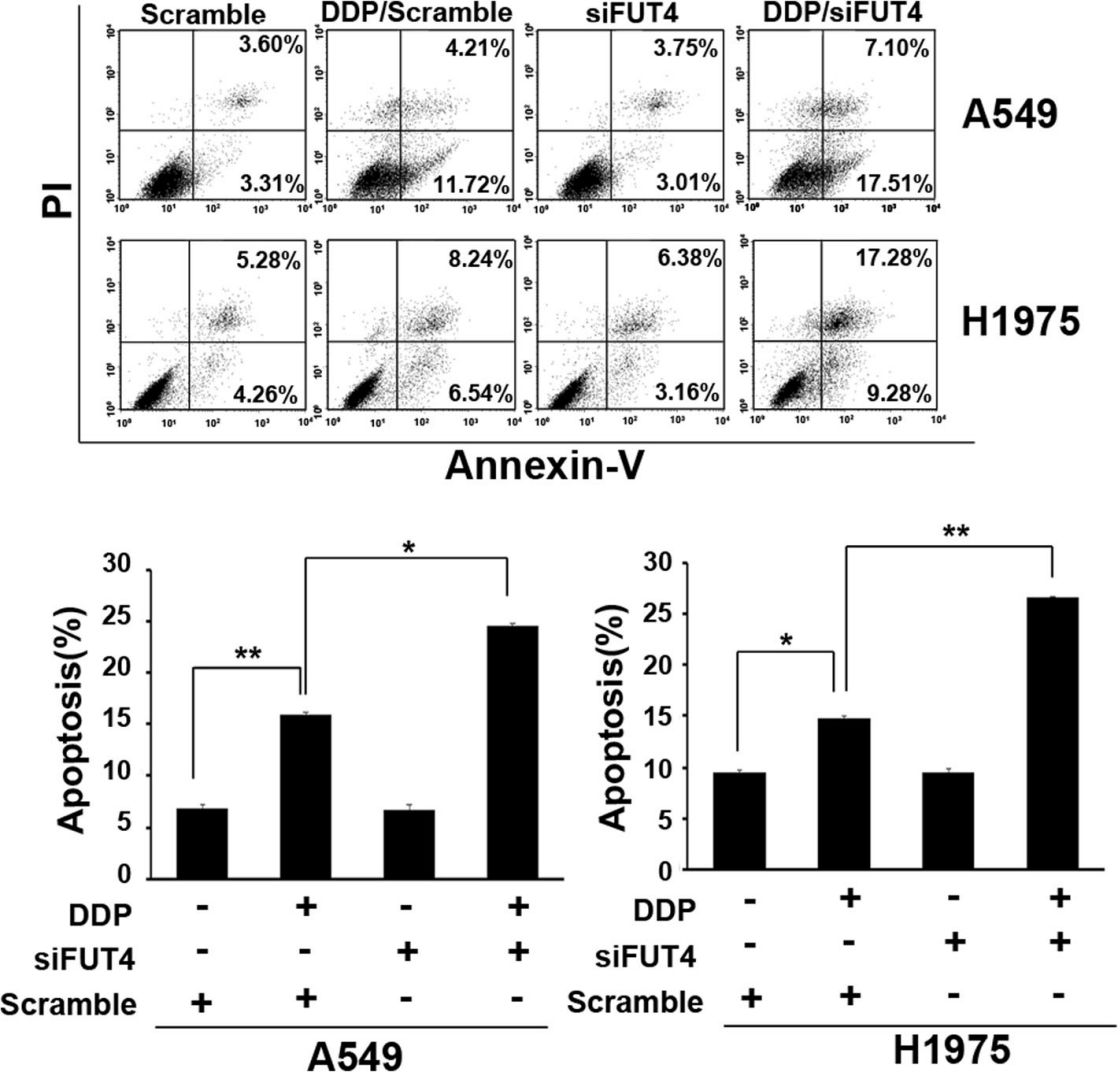
Potential role of FUT4 in cisplatin-induced cell apoptosis in A549 and H1975 cells. Flow cytometry was applied to detect cell apoptosis after A549 and H1975 cell transfection with FUT4siRNA or si-NC, followed by treatment with cisplatin (3 μg/ml). FUT4siRNA combined with cisplatin significantly induced apoptosis in A549 and H1975 cells. Data are mean ± SD from 3 independent experiments. *P < 0.05, **P < 0.01

To examine whether growth suppression in cells resulted from enhanced apoptosis, we assessed cell apoptosis by flow-cytometry. The results showed that FUT4siRNA markedly increased cisplatin’s cytotoxicity towards A549 and H1975 cells. The proportions of A549 and H1975 cells undergoing apoptosis were significantly increased following cisplatin treatment compared with control cells (*P* < 0.05 and *P* < 0.01, respectively; Fig. 2). Notably, FUT4siRNA transfection further increased apoptotic rates in presence of cisplatin (*P <* 0.05; Fig. 2).

**Fig. 3:**
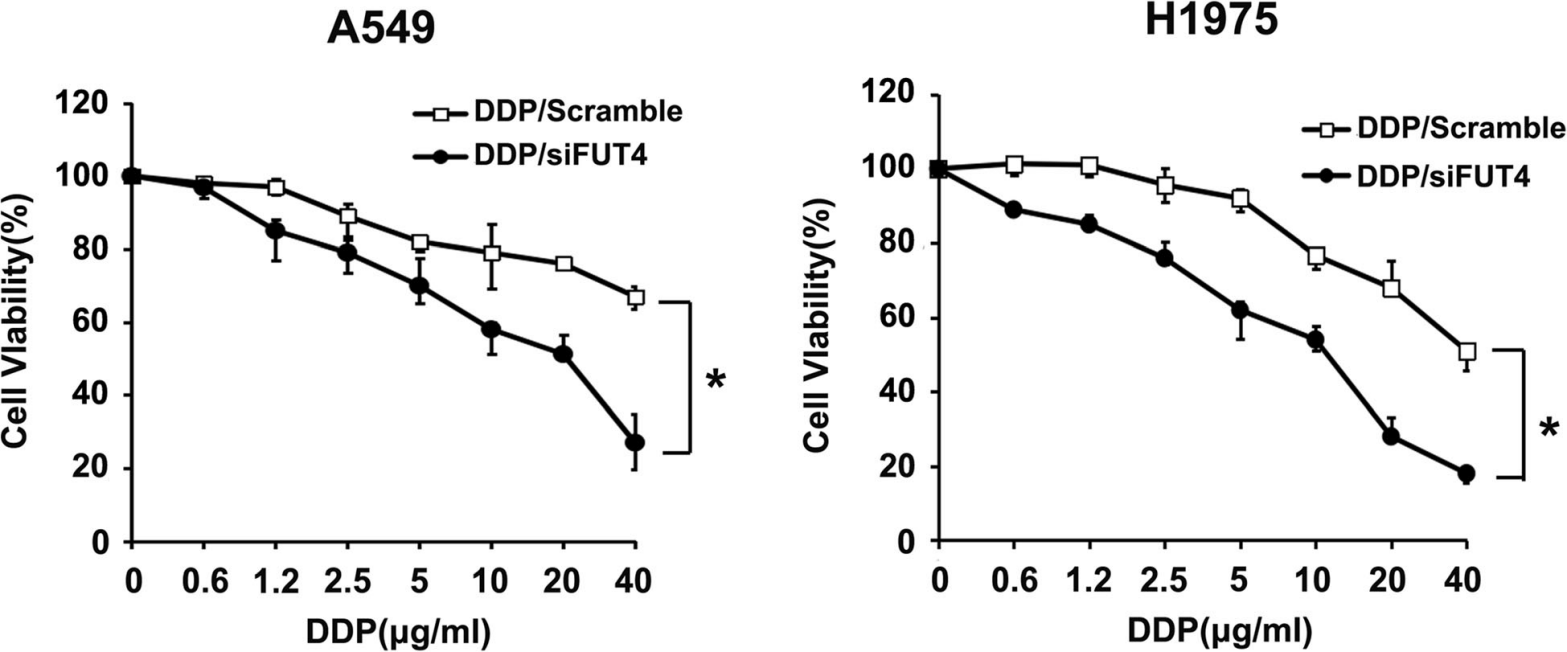
FUT4siRNA transfection increases the chemosensitivity of A549 and H1975 cells to cisplatin. CCK-8 assay was applied to detect cell viability after A549 and H1975 cells were transfected with FUT5siRNA or si-NC, followed by treatment with various concentrations of cisplatin (0, 0.6, 1.2, 2.5, 5, 10, 20, 40 and 40 μg/ml). Data are mean ± SD from 3 independent experiments. *P < 0.05

To demonstrate the importance of the FUT4 gene in lung carcinoma chemosensitivity to cisplatin, FUT4 siRNA was generated. Figure 1a shows that administration of FUT4-siRNA reduced FUT4 protein amounts at 48 h following transfection. Control and FUT4-siRNA transfected A549 and H1975 cells were administered cisplatin in various amounts, and CCK-8 assay was used to assess cell viability. As shown in Fig. 3, FUT4 silencing dose-dependently altered cell viability in comparison with the control group, Fig. 3 showed that siRNAs against FUT4 significantly decreased cellular viability.

### Silencing of FUT4 expression reduces the proliferation, invasion and migration of A549 and H1975 cells with cisplatin treatment

**Fig. 4:**
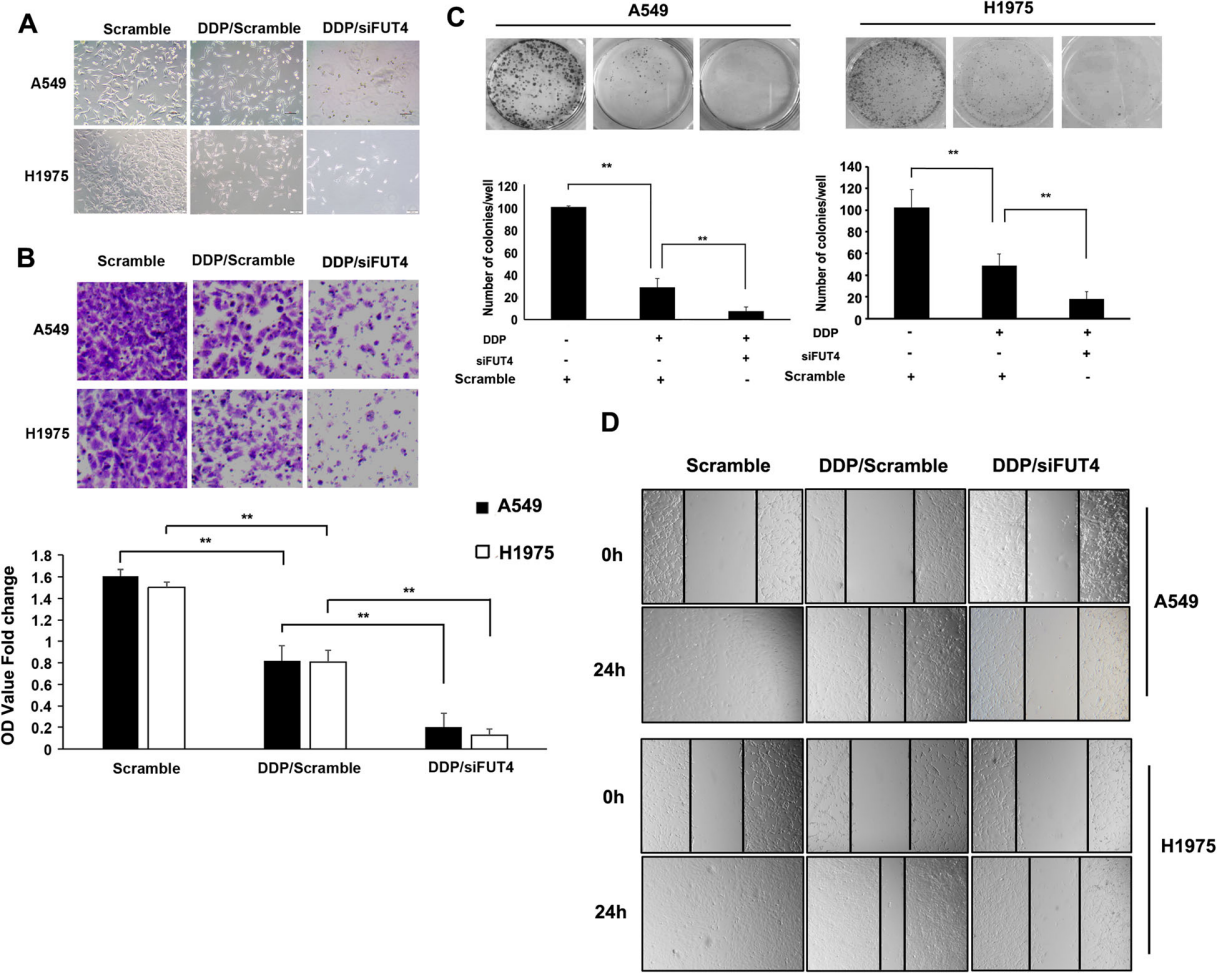
FUT4 mediates the inhibition of cell growth, invasion, migration and colony formation in A549 and H1975 cells treated with cisplatin. **a** FUT4siRNA combined with cisplatin significantly inhibited cell growth. **b** FUT4siRNA in combination with cisplatin significantly inhibited the invasive abilities of A549 and H1975 cells. **c** Colony formation abilities of A549 and H1975 cells were observed after cisplatin treatment following transfection with FUT4siRNA or si-NC. A549 and H1975 cell colonies were stained with crystal violet. **d** Cell migration ability of A549 and H1975 cells were detected by wound healing assay

Next, FUT4siRNA combination with cisplatin (3 μg/ml) was evaluated for its effects on proliferation, invasion and migration ability in A549 and H1975 cells, respectively. The results revealed that treatment with cisplatin alone reduced proliferation (*P* < 0.01; Fig. 4a & c), invasion (*P* < 0.05; Fig. 4b) and migration (Fig. 4d) in A549 and H1975 cells compared with control cells. However, FUT4siRNA/cisplatin combination was more potent in reducing cell proliferation (*P <* 0.01; Fig. 4a & c), invasion (*P <* 0.01; Fig. 4b) and migration (Fig. 4d) in A549 and H1975 cells compared with cisplatin treatment alone. These findings suggested FUT4 played an important role in cisplatin-mediated chemosensitivity of NSCLC cells by suppressing colony formation and invasion in cancer cells.

### FUT4 silencing sensitizes A549 and H1975 cells to cisplatin via inactivation of EGFR/PI3K/AKT signaling and FOXO1 phosphorylation

**Fig. 5:**
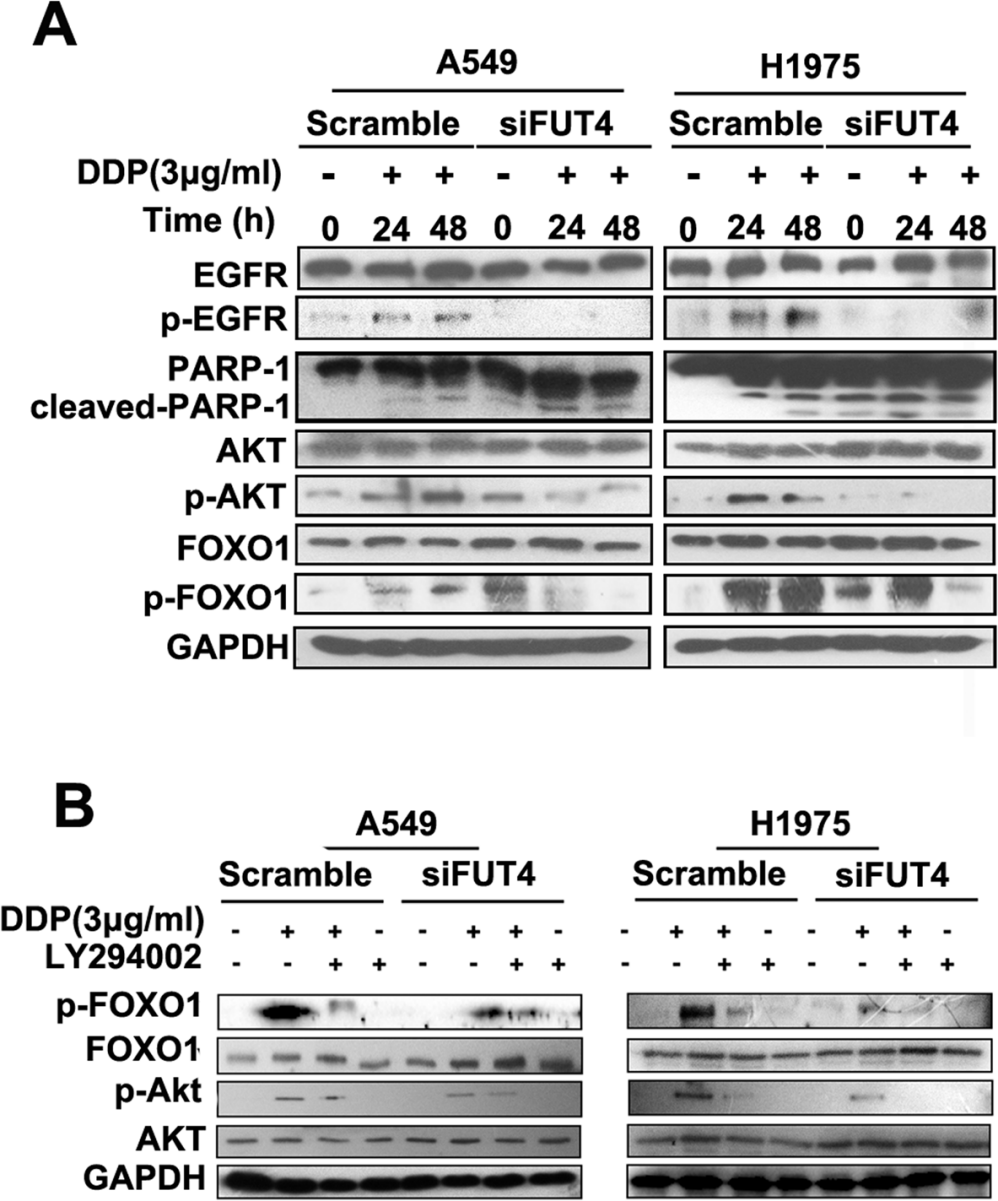
Involvement of FUT4 in cisplatin-induced EGFR/Akt signaling. **a** si-NC and FUT4siRNA-infected cells were treated with cisplatin (3 μg/ml) for up to 48 h, and cell lysates were subjected to 12% SDS-PAGE to measure the levels of phosphorylated EGFR, Akt and FOXO1. The membranes used for anti-phospho antibody staining were stripped and reused for total EGFR, Akt and FOXO1 levels, respectively. GAPDH was used as an internal control. **b** Effects of pharmacological inhibition of Akt on cisplatin-induced phosphorylation of FOXO1. Cells were pre-incubated with or without the PI3K inhibitor LY294002 and further incubated in the presence or absence of cisplatin (3 μg/ml). Cell lysates were used to measure FOXO1 and Akt phosphorylation levels

We further examined how EGFR/PI3K/Akt signaling and FOXO1 phosphorylation participate in the control of cisplatin-induced apoptosis in FUT4siRNA transfected A549 and H1975 cells. Cisplatin administration caused a time-dependent increase in EGFR and Akt phosphorylation levels in scrambled-siRNA treated A549 and H1975 cells. Meanwhile, markedly decreased EGFR and Akt phosphorylation levels were observed at 24 h and 48 h following cisplatin treatment of FUT4siRNA transfected A549 and H1975 cells (Fig. 5a). In addition to assessing EGFR/PI3K/Akt induction, the potential modulatory effect of FOXO1 on apoptosis was evaluated in A549 and H1975 cells. The results showed that FUT4 silencing reduced cisplatin-induced FOXO1 phosphorylation (Fig. 5a).

To explore how Akt activation affects cell apoptosis induced by cisplatin, the PI3K inhibitor LY294002 was assessed for its effects on scrambled- and FUT4siRNA transfected A549 and H1975 cells. First, LY294002 or the vehicle was administered to cells, followed by treatment with cisplatin (3 μg/ml). As shown in Fig. 5b, cisplatin treatment augmented Akt and FOXO1 levels, while LY294002 reduced these effects in scrambled-siRNA transfected A549 and H1975 cells. Meanwhile, more remarkable inhibition of cisplatin-induced activation of Akt and FOXO1 by LY294002 was observed in FUT4siRNA transfected cells.

### FUT4 modulates nuclear translocation of the FOXO1 protein

**Fig. 6:**
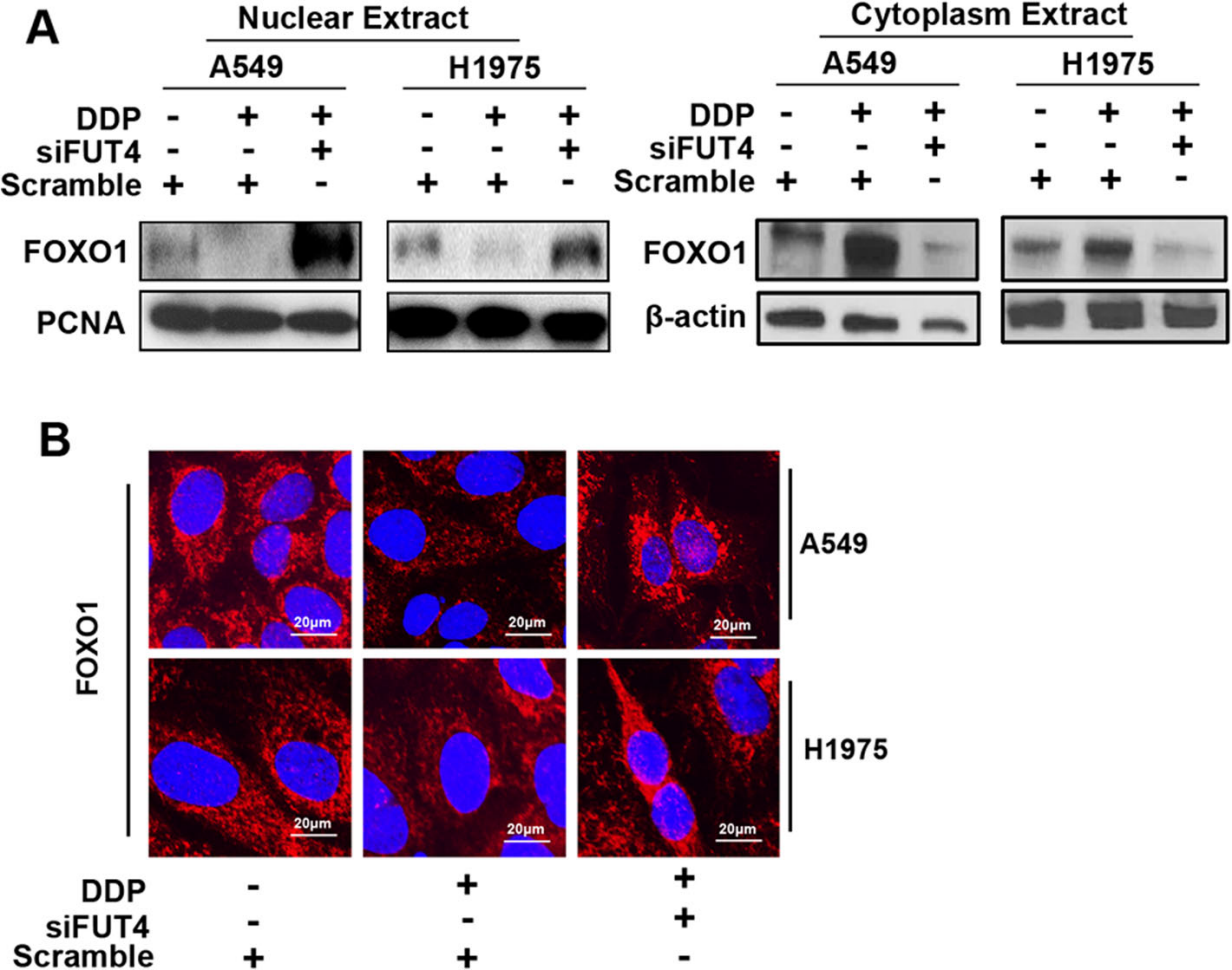
FUT4siRNA affects FOXO1 translocation. **a** Nuclear and cytoplasm extracts from A549 and H1975 cells were subjected to 12% SDS-PAGE to measure the protein levels of FOXO1. **b** Confocal microscopy for FoxO1 detection in A549 and H1975 cells. Panels show merged DAPI and FoxO1 signals obtained with/without FUT4 knockdown in A549 and H1975 cells with/without cisplatin treatment

The effect of FUT4 knockdown on protein expression of the transcription factor FOXO1 was assessed by Western blot (Fig. 6a) and immunofluorescence (Fig. 6b), detecting FOXO1 in nuclear fractions.

Immunofluorescent staining and immunoblot were employed to assess FOXO1 localization and amounts in A549 and H1975 cells after cisplatin and/or FUT4siRNA treatments. As shown in Fig. 6 FUT4siRNA combined with cisplatin treatment significantly promoted FOXO1 expression in the nucleus of A549 and H1975 cells.

## Discussion

Cisplatin is a widely used chemotherapeutic drug for treating various solid tumors, including NSCLC [20]. The efficiency of cisplatin-based combination therapy can only be sustained for a short time [21]. Here, the mechanism by which FUT4 regulates the chemosensitivity of NSCLC cells to cisplatin was explored, and the interplay between FUT4 and cisplatin induced EGFR/PI3k/AKT pathway activation was demonstrated. Indeed, knockdown of FUT4 significantly augmented the inhibitory effects of cisplatin on tumor growth.

Specific changes of cell surface molecules can be detected during cell apoptosis, proliferation and chemoresistance, including the altered expression of oligosaccharide chains in some specific proteins [22]. Via catalysis of Fuc moiety transfer from GDP-Fuc to respective oligosaccharide acceptors in 1, 2- (FUT1 and FUT2), a1, 3/4- (FUT3, FUT4, FUT5, FUT6, FUT7, FUT9, FUT10 and FUT11) and 1, 6- (FUT8) linkages, FUTs facilitate the biosynthesis of fucosylated oligosaccharide chains in glycoconjugates [23–25]. Among these, the FUT4 gene plays a critical role in regulating tumor chemosensitivity. For example, FUT4 knockdown increases BEL/FU cell chemosensitivity [10]. Meanwhile, FUT4 upregulation contributes to drug resistance in human HCC BEL7402 and BEL/FU cells [10]. In addition, transcriptional upregulation of FUT4, which increases cell-surface Lewis Y antigen levels, leads to chemoresistance in patients with colorectal cancer [26]. The present study demonstrated that FUT4 gene silencing significantly increased the suppressive effects of cisplatin on cancer cell proliferation and boosted apoptosis. These finding suggested that FUT4 might have a critical function in regulating NSCLC chemosensitivity to cisplatin.

The PI3K/Akt signaling pathway plays a critical role in controlling the levels and functions of proteins required for chemoresistance of cancer cells [27, 28]. Inhibition of PI3K/Akt signaling can effectively augment tumor cell chemosensitivity to cisplatin [29, 30]. The correlation between FUT4 and PI3K/Akt pathway activation has been demonstrated, suggesting FUT4-modulated HCC cell ADR is, to some extent, PI3K/Akt-dependent [10]. Further assessment showed that overexpression of FUT4 promotes the proliferation of A431 cells by activating the PI3K/Akt pathway [9]. the above results indicated that suppression of the FUT4 gene augmented NSCLC chemosensitivity to cisplatin through blocking of PI3K/Akt signaling. We also explored the mechanism by which FUT4 knockdown inhibits PI3K/Akt. As shown above, FUT4 silencing significantly inhibited cisplatin induced EGFR activation. Upon ligand binding, EGFR forms homo- and heterodimers that induce several downstream pathways, including PI3K/Akt signaling [31, 32]. In addition, anti-Lewis Y antibody (IGN311) effectively blocks EGF-induced MAPK phosphorylation by suppressing the EGFR pathway in A431 cells [33]. The above results demonstrated that FUT4 knockdown directly downregulated Lewis Y antigen synthesis, indicating that inhibition of FUT4 blocks PI3K/Akt activation by attenuating Lewis Y antigen dependent EGFR phosphorylation, thus enhancing the chemosensitivity of NSCLC cells to cisplatin.

FOXO1 belongs to the FOXO family of transcription factors (FoxOs). The major types of malignant tumors show FOXO1 inactivation by hyperactivation of PI3K/Akt signaling [34]. FOXO1 has tight association with death-receptor-mediated apoptosis, and Akt directly inactivates FOXO1, downregulating FOXO1-controlled proteins, which are closely related to cell apoptosis [35]. Upon phosphorylation by Akt, FOXO1 undergoes translocation from the nucleus to the cytoplasm, losing its transcriptional activity. Gao and colleagues reported that FOXO1 contributes to Mirk-mediated cell survival and ovarian cancer chemosensitivity to cisplatin [36]. Park Jinju et al reported that FOXO1 mediates cisplatin resistance in gastric cancer cells [37]. Inhibition of FOXO1 nuclear export restores sensitivity to AKT-associated erlotinib resistance in lung cancer cells [36]. In the present study, FUT4 knockdown augmented FOXO1 dephosphorylation by inactivating EGFR/PI3K/Akt signaling. Furthermore, we found that FOXO1 was re-translocated to the nucleus, recovering its transcriptional activity, which augments cisplatin induced cell apoptosis. These findings suggest that FOXO1 might constitute a mediator of the increased apoptosis observed in FUT4siRNA transfected cells treated with cisplatin. Further studies are required to elucidate the histopathological examinations of sampled xenotransplanted tumors in order to certify the exact mechanism by which FUT4siRNA increase the chemosenstivity of NSCLC to cisplatin.

## Conclusion

**Fig. 7:**
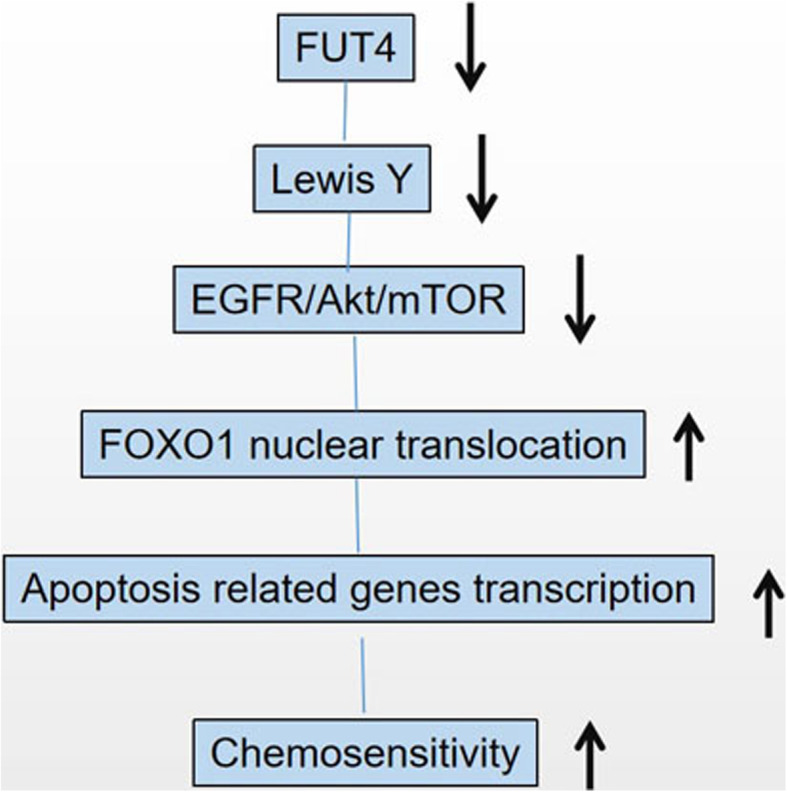
Mechanism of FOXO1 regulation via FUT4/LeY expression

FUT4 augments NSCLC chemosensitivity to cisplatin by controlling the transcriptional activity of FOXO1. The mechanistic detail is shown in Fig. 7, which helps to state the novel idea of this paper comprehensively. These findings provide evidence supporting the application of combination of cisplatin and FUT4 inhibitors in treating lung carcinoma in the future. FUT4 may constitute an effective sensitization target in developing cisplatin-based chemotherapies for lung carcinoma.

## Data Availability

The authors declare that the materials included in this manuscript, including all relevant raw data, may be made freely available to any researchers who wish to use them for non-commercial purposes, while preserving any necessary confidentiality and anonymity.

## Abbreviations

FUT4: Fucosyltransferase IV
NSCLC: Non-small cell lung cancer
FUTs: Fucosyltransferases
FOX: O-class forkhead factors
DDP: Cisplatin
FBS: Fetal bovine serum
RIPA: Radio-immunoprecipitation assay
SDS-PAGE: SDS polyacrylamide gel electrophoresis
LeY: Lewis Y
